# The EHR Density Index: A new method to control for EHR data inconsistency across patients

**DOI:** 10.64898/2026.08.03.26359595

**Published:** 2026-08-06

**Authors:** Abhishek Bhatia, Sydney Lash, Tomas McIntee, Emily R. Pfaff

## Abstract

Electronic health record (EHR) data vary substantially in documentation density across patients, independent of disease burden. Existing tools such as the Charlson Comorbidity Index (CCI) and Elixhauser Comorbidity Index measure disease burden but do not capture differences in data volume, leaving a common source of bias unaddressed in EHR-based analyses. To address this gap, we developed the EHR Density Index (EDI), which characterizes the quantity, depth, and breadth of EHR data per patient per year, normalized by utilization patterns, using records from 24,987 adult patients at UNC Health (2018–2024). The EDI combines a utilization cluster assigned via Gaussian Mixture Model with within-cluster residuals quantifying documentation volume across four clinical domains. Four interpretable clusters emerged; while CCI predicted cluster membership, its associations with within-cluster residuals were weak, confirming the EDI captures dimensions of the patient record distinct from disease burden. The EDI is intended as a covariate to address documentation density as a source of confounding in real-world data-driven research.

## Full Text Availability

The license terms selected by the author(s) for this preprint version do not permit archiving in PMC. The full text is available from the preprint server.

